# A Specific Host/Microbial Signature of Plasma-Derived Extracellular Vesicles Is Associated to Thrombosis and Marrow Fibrosis in Polycythemia Vera

**DOI:** 10.3390/cancers13194968

**Published:** 2021-10-02

**Authors:** Martina Barone, Monica Barone, Francesca Ricci, Giuseppe Auteri, Francesco Fabbri, Erika Bandini, Francesco Francia, Pier Luigi Tazzari, Nicola Vianelli, Silvia Turroni, Michele Cavo, Lucia Catani, Marco Candela, Francesca Palandri

**Affiliations:** 1 Istituto di Ematologia “Seràgnoli”, Department of Experimental, Diagnostic and Specialty Medicine, University of Bologna,40138 Bologna, Italy; martina.barone5@unibo.it (M.B.); giuseppe.auteri2@unibo.it (G.A.); michele.cavo@unibo.it (M.C.); 2 Department of Medical and Surgical Sciences, University of Bologna, 40138 Bologna, Italy; monica.barone@unibo.it; 3 Department of Pharmacy and Biotechnology, University of Bologna, 40138 Bologna, Italy; francesco.francia@unibo.it (F.F.); silvia.turroni@unibo.it (S.T.); marco.candela@unibo.it (M.C.); 4 Servizio di Immunoematologia e Trasfusionale, IRCCS Azienda Ospedaliero-Universitaria di Bologna, 40138 Bologna, Italy; francesca.ricci@aosp.bo.it (F.R.); pierluigi.tazzari@aosp.bo.it (P.L.T.); 5 IRCCS Istituto Romagnolo per lo Studio dei Tumori (IRST) “Dino Amadori”, 47014 Meldola, Italy; francesco.fabbri@irst.emr.it (F.F.); erika.bandini@irst.emr.it (E.B.); 6 Istituto di Ematologia “Seràgnoli”, IRCCS Azienda Ospedaliero-Universitaria di Bologna, 40138 Bologna, Italy; nicola.vianelli@unibo.it (N.V.); francesca.palandri@unibo.it (F.P.)

**Keywords:** polycythemia vera, extracellular vesicles, microbial DNA cargo, biomarker, thrombosis, marrow fibrosis

## Abstract

**Simple Summary:**

Patients with polycythemia vera, a myeloproliferative neoplasm, are at increased risk of thrombosis and progression to myelofibrosis. However, no disease-specific risk factors have been identified so far. Extracellular vesicles, released from a broad variety of cells, are receiving increasing attention for their effects on cell-to-cell communication. In addition, they play a role in cancer and thrombosis. Interestingly, circulating microbial components/microbes have been recently indicated as potential modifiers of inflammation and coagulation. Here, we identified a signature of thrombosis history and marrow fibrosis by analyzing the phenotype and the microbial DNA cargo of the circulating extracellular vesicles after isolation from the plasma of patients with polycythemia vera. These data may support the role of extracellular vesicles as liquid biomarkers of aggressive disease, thus contributing to refining the prognosis of polycythemia vera.

**Abstract:**

Polycythemia vera is a myeloproliferative neoplasm with increased risk of thrombosis and progression to myelofibrosis. However, no disease-specific risk factors have been identified so far. Circulating extracellular vesicles (EVs) are mostly of megakaryocyte (MK-EVs) and platelet (PLT-EVs) origin and, along with phosphatidylethanolamine (PE)-EVs, play a role in cancer and thrombosis. Interestingly, circulating microbial components/microbes have been recently indicated as potential modifiers of inflammation and coagulation. Here, we investigated phenotype and microbial DNA cargo of EVs after isolation from the plasma of 38 patients with polycythemia vera. Increased proportion of MK-EVs and reduced proportion of PLT-EVs identify patients with thrombosis history. Interestingly, EVs from patients with thrombosis history were depleted in *Staphylococcus* DNA but enriched in DNA from Actinobacteria members as well as *Anaerococcus*. In addition, patients with thrombosis history had also lower levels of lipopolysaccharide-associated EVs. In regard to fibrosis, along with increased proportion of PE-EVs, the EVs of patients with marrow fibrosis were enriched in DNA from *Collinsella* and *Flavobacterium*. Here, we identified a polycythemia-vera-specific host/microbial EV-based signature associated to thrombosis history and marrow fibrosis. These data may contribute to refining PV prognosis and to identifying novel druggable targets.

## 1. Introduction

Polycythemia vera (PV) is a Philadelphia-negative myeloproliferative neoplasm (MPN) characterized by clonal myeloproliferation and expansion of the erythrocyte mass. PV pathogenesis relies on hyperactivation of the *JAK2* gene, driven by *JAK2V617F* (95% of cases) or exon 12 mutation (3% of cases). However, in 2% of cases, no driver mutation can be detected [1,2]. Clinical phenotype includes systemic symptoms, splenomegaly, progression to post-PV myelofibrosis (PPV-MF), and increased risk of thrombosis. Known risk factors for progressive disease are older age, leukocytosis, and a complex karyotype [3,4]. Recently, it has been demonstrated that additional gene mutations besides *JAK2* and *TET2* confer an increased risk for fibrotic progression in PV [5,6].

Thrombosis may occur prior to or at the time of diagnosis and/or after diagnosis [7,8]. Conventional risk assessment relies upon two patient-related features, i.e., age > 60 years and/or previous thrombosis. The risk of thrombosis is increased 5–10-fold in high-risk (≥1 risk factor) and 2–4-fold in low-risk patients [9,10]. In addition, leukocytosis and cardiovascular risk factors also identify patients at higher thrombotic risk [7]. However, conventional risk factors are not PV-specific. In addition, only *JAK2V617F* variant allele frequency (VAF) > 50% has been associated with a higher thrombotic risk and bone marrow fibrosis in PV [11,12]. Aggressive hematocrit control <45% and use of cytoreductive treatment when required have been shown to reduce the number of thrombotic events and substantially improve survival [8]. However, thrombosis still remains a major cause of illness and death [13].

Accumulating evidence is revealing possible links between cardiovascular disorders and dysbiosis of the gut microbiota [14,15]. In parallel, few recent works have shown the presence of circulating microbial factors (e.g., lipopolysaccharide (LPS)) and microbes, which could directly affect hemopoiesis [16,17,18]. Though contamination phenomena cannot be completely ruled out, these blood microbial components are supposed to be present even in healthy individuals, probably derived by human-associated microbial communities (primarily, intestine, mouth and skin) [19,20].

Extracellular vesicles (EVs), released from a broad variety of eukaryotic as well as prokaryotic cells, are receiving increasing attention for their effects on (interkingdom) cell signaling [21,22]. Most circulating EVs are of megakaryocyte (MK-EVs) and platelet (PLT-EVs) origin. Human-cell-derived EVs, including PLT-EVs, phosphatidylethanolamine- and tissue-factor-positive EVs (PE-/TF-EVs), are involved in cancer and thrombotic disorders [23,24,25,26]. On the other hand, less is known about the impact of bacterial EVs on human physiology. It has recently been suggested that they can influence the functioning of the immune system through direct activation of host receptors, delivery of bacterial content, or incorporation into the host cell cytoplasm [27].

In this study, we aimed to identify a “signature” of outcome in PV patients by analyzing the phenotype and microbial DNA cargo of circulating EVs and by investigating the gut microbiota composition. Our data provide evidence that a distinct phenotype and microbial DNA layout characterize the EVs of patients with thrombosis history or marrow fibrosis.

## 2. Materials and Methods

### 2.1. Study Cohort

The “EV-PV” study is a monocenter clinical biological study promoted by the Institute of Hematology “L. e A. Seràgnoli”, S. Orsola-Malpighi Bologna University Hospital, and performed in collaboration with the Department of Pharmacy and Biotechnology, University of Bologna.

Thirty-eight PV patients (35 JAK2V617F- and 3 Exon12-mutated) were enrolled. Patient clinical/laboratory characteristics are shown and described in Table S1. PV diagnosis was assessed according to WHO 2016 criteria [28]. PV patients were treated according to standard clinical practice and were retrospectively evaluated for thrombotic events and other outcome parameters (bleedings, infections, second neoplasia, evolution into post-PV myelofibrosis or acute leukemia, death). Exclusion criteria were acute illness and antibiotic treatment for two months preceding the study. Thrombotic events were objectively identified based on diagnostic imaging. Only major thrombotic events were considered (arterial thromboses, acute myocardial infarction, angina pectoris, ischemic stroke, transient ischemic attack, peripheral arterial disease; venous thromboses: pulmonary embolism, deep vein thrombosis, portal or mesenteric thrombosis, cerebral sinus vein thrombosis). Thromboses after diagnosis were defined as events occurring >4 weeks from diagnosis. Marrow fibrosis categories were identified following the WHO 2016 criteria.

### 2.2. Collection of Peripheral Blood and Fecal Samples and Platelet-Poor Plasma Preparation

EDTA-anticoagulated peripheral blood and fecal samples were collected from PV patients. After discarding the first 2 mL of blood, platelet-poor plasma (PPP) was obtained (within 2 h from blood collection) after two consecutive centrifugations at 2500× *g* for 15 min at room temperature. PPP was then aliquoted and stored at −80 °C until testing. Fecal samples were immediately frozen at −20 °C and then delivered to the Unit of Microbial Ecology of Health (Department of Pharmacy and Biotechnology, University of Bologna, Bologna, Italy), where they were stored at −80 °C until processing.

### 2.3. Phenotype Analysis of Plasma EVs

Circulating MK (CD61+/CD62P−), PLT (CD61+/CD62P+), TF (CD142+)−, and PE (duramicin+)− EVs were analyzed by flow cytometry (Navios, Beckman Coulter, Milan, Italy) in PPP, after thawing at 37 °C, as previously described [29]. Briefly, PPP (100 µL) was incubated at 4 °C for 15 min with antibodies or PE-specific probe (duramycin), then diluted 1:3 and acquired immediately (list of monoclonal antibodies and reagents according to EV subtype is shown in Table S2). To detect EVs, the instrument was calibrated with MegaMix Beads (Stagò, Marseille, France). Fluorescence-gated polystyrene beads of different sizes were used to determine the gates identifying big (500–900 nm), small (200–300 nm), and nano (100–160 nm) EVs, as previously described [29]. The violet side scatter laser (VSSC) was used as a trigger signal to discriminate noise. Our analysis was focused on big EVs in order to avoid unspecific staining (i.e., debris); notably, we were unable to find any significant difference between the groups when smaller EVs were investigated. Big EVs were identified by using the size and ability to bind specific monoclonal antibodies. Matched isotype controls were used to select the cut-off. By using the defined gate for big EVs, all events positive for marker staining were recorded and expressed as a percentage of positive EVs.

### 2.4. Enumeration and Phenotype Analysis of EVs after Isolation from the Plasma

EVs were isolated from thawed PPP (2 mL at 37 °C) by ultracentrifugation at 100,000× *g* for 2 h at 4 °C with Optima L-90k ultracentrifuge (Beckman Coulter) equipped with Type 90Ti rotor, as previously described [30]. EVs pellets were then resuspended in twice filtered (filter pore size, 0.22 µm) RPMI supplemented with 1% DMSO and stored at −80 °C until use. EV enumeration was assessed by using the NanoSight technology (NanoSight NS300—Malvern Panalytical Ltd., Royston, UK) and nanoparticle tracking analysis software (NTA 2.3 Proprietary Software—Malvern Panalytical Ltd., Royston, UK). The phenotype of isolated EVs was characterized by flow cytometry (Navios, Beckman Coulter, Milan, Italy) using monoclonal antibodies against tetraspanins (CD9, CD63, CD81), CD61, CD62P, TF (CD142) and lipopolysaccharide (LPS)- or PE-specific probes (Duramycin) (list of monoclonal antibodies and reagents according to EVs subtype is shown in Table S2). Conjugated mouse isotypic IgG was used as a control. Briefly, EVs (2 × 108) were stained at 4 °C for 15 min, then diluted 1:3 and acquired immediately. Tetraspanins expression was analyzed on total isolated EVs, while the other markers were analyzed on big EVs.

### 2.5. Microbial DNA Extraction from Feces and Isolated EVs and 16S rRNA Gene Amplicon Sequencing

Microbial DNA was extracted from feces (250 mg) and isolated EVs (2 mL of PPP) using the repeated bead-beating plus columns method, as previously described with a few modifications [31]. In brief, all samples were suspended in 1 mL of lysis buffer (500 mM NaCl, 50 mM Tris-HCl pH 8, 50 mM EDTA, and 4% (*w/v*) SDS), added to four 3 mm glass beads and 0.5 g of 0.1 mm zirconia beads (BioSpec Products, Bartlesville, OK, USA) and bead-beaten in a FastPrep instrument (MP Biomedicals, Irvine, CA, USA) at 5.5 movements/s for 1 min. Only one homogenization step was performed for EVs samples, while for stool samples it was repeated three times, incubating the samples on ice for 5 min between treatments. After incubation at 95 °C for 15 min, all samples were centrifuged at 13,000 rpm for 5 min. Nucleic acids were precipitated by adding 260 µL of 10 M ammonium acetate and one volume of isopropanol. The pellets were then washed with 70% ethanol and suspended in 100 µL of TE buffer (10 mM Tris-HCl, 1 mM EDTA, pH 8). RNA was removed by treatment with 2 µL of 10 mg/mL DNase-free RNase at 37 °C for 15 min. Protein removal and column-based DNA purification were performed by using the DNeasy Blood and Tissue kit (Qiagen, Hilden, Germany) and following the manufacturer’s instructions. Template-free controls (i.e., RPMI medium and extraction kit reagents) were processed as well, in the same way as the samples. DNA was quantified with the NanoDrop ND-10000 spectrophotometer (NanoDrop Technologies, Wilmington, DE, USA).

### 2.6. 16S rRNA Gene Amplification and Sequencing

The V3–V4 hypervariable region of the 16S rRNA gene was amplified from DNA extracted from isolated EVs, stool samples, and no-template controls by using the 341F and 785R primers with Illumina overhang adapter sequences, as previously described by Klindworth et al. [32]. PCR reactions were performed by using KAPA HiFi HotStart ReadyMix (Roche, Mannheim, Germany), in a Thermal Cycler T (Biometra, Göttingen, Germany) with the following gradient: 3 min at 95 °C for the initial denaturation, 25 cycles of denaturation at 95 °C for 30 s, annealing at 55 °C for 30 s, elongation at 72 °C for 30 s, and a final elongation step at 72 °C for 5 min. PCR products of around 460 bp were purified using a magnetic bead-based system (Agencourt AMPure XP, Beckman Coulter), and a limited-cycle PCR using Nextera Technology was performed to obtain the indexed library, followed by a second clean-up step as described above. Indexed libraries were pooled at an equimolar concentration of 4 nM, denatured with NaOH 0.2 N, and diluted to 5 pM before loading onto the Illumina MiSeq flow cell. The 2 × 250 bp paired-end sequencing protocol was performed according to the manufacturer’s instructions (Illumina, San Diego, CA, USA). Sequencing reads were deposited in the National Center for Biotechnology Information Sequence Read Archive (NCBI SRA) under the following Bioproject number: PRJNA737425 (https://www.ncbi.nlm.nih.gov/bioproject/PRJNA737425).

### 2.7. Bioinformatics and Biostatistics

Raw sequences were processed using a pipeline that combines PANDAseq [33] and QIIME 2 [34]. After length (min/max = 350/500 bp) and quality filtering (default parameters), cleaned reads were binned into amplicon sequence variants (ASVs) using DADA2 [35,36]. Taxonomy was assigned through the VSEARCH algorithm [37], using the Greengenes database as a reference (release May 2013). All singleton ASVs were discarded. Moreover, for EVs, ASVs were considered putative contaminants and, therefore, removed when their mean relative abundance did not exceed 20% of that in controls, similarly to what was previously applied by Dash and colleagues [38]. Alpha diversity was evaluated using two different metrics: Shannon and inverse Simpson (1/D). The Jaccard similarity index was used to construct principal coordinates analysis (PCoA) plots.

All statistical analyses were performed in R 3.6.1, using R Studio 1.2.1335 and the vegan [39], made4 [40], and stats [41] packages. The significance of data separation in the PCoA was tested by means of PERMANOVA (adonis function of the vegan package). Wilcoxon rank-sum test was used to assess significant differences between groups (for intra- and inter-individual diversity as well as taxon relative abundance), while Kruskal–Wallis test was used for multiple comparisons. *p*-values were corrected for multiple testing using the Benjamini–Hochberg method, and a false discovery rate (FDR) ≤ 0.05 was considered statistically significant.

As for the phenotype of EVs, statistical analysis was performed with GraphPad (GraphPad Software Inc., La Jolla, CA, USA). The differences between the groups were analyzed with Mann–Whitney, Chi-square, or Spearman’s correlation tests, as appropriate. *p*-values were considered significant when ≤0.05 (2-tailed).

## 3. Results

### 3.1. Circulating EVs from PV Patients Carry a Distinctive Thrombosis/Marrow Fibrosis-Related Phenotype

**Figure 1:**
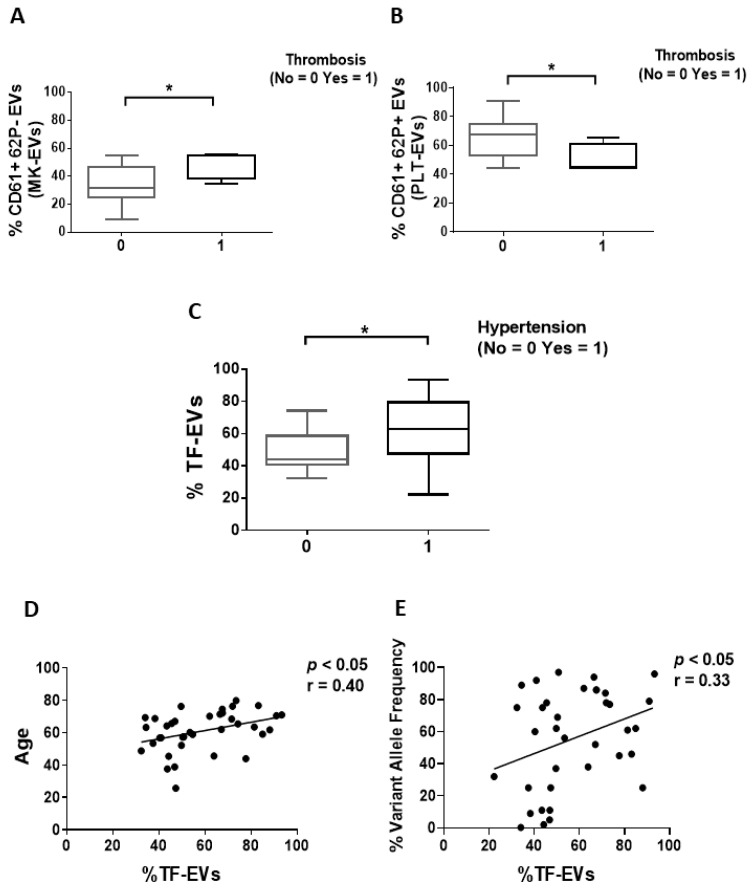
Proportion of circulating MK-, PLT-, and TF-EVs of PV patients. (**A**,**B**) Frequency of MK- and PLT-EVs in PV patients with (*n* = 14) or without thrombosis history (*n* = 24). (**C**) Frequency of TF-EVs of PV patients with (*n* = 24) or without (*n* = 14) hypertension. (**A**–**C**) Data are expressed as percentage of MK-, PLT-, and TF-EVs and presented as min to max with median (Mann–Whitney test; * *p* < 0.05). (**D**,**E**) TF-EV percentages of PV patients positively correlate with age and JAK2V617F-variant allele frequency (Spearman’s correlation test).

**Figure 2:**
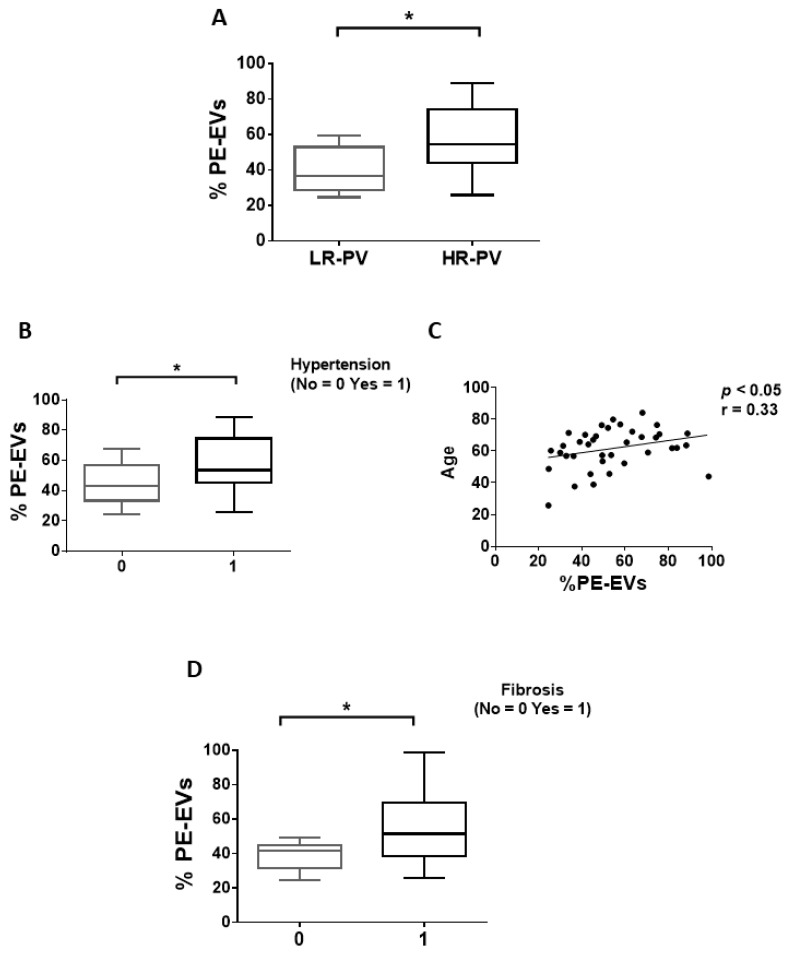
Proportion of circulating PE-EVs of PV patients. (**A**) Frequency of PE-EVs in PV patients with low (LR-PV; *n* = 11) vs. high (HR-PV; *n* = 27) risk. (**B**) Proportion of PE-EVs of PV patients with (*n* = 24) or without (*n* = 14) hypertension. (**C**) PE-EVs percentages of PV patients positively correlate with age (Spearman’s correlation test). (**D**) Frequency of PE-EVs in PV patients with (*n* = 10) or without (*n* = 28) marrow fibrosis. (**A**,**B**,**D**) Data are expressed as percentage of PE-EVs and presented as min to max with median (Mann–Whitney test; * *p* < 0.05).

We firstly compared the EV phenotype of PV patients with or without thrombosis or marrow fibrosis. Patients with thrombosis history showed significantly increased and decreased proportion of MK-EVs and PLT-EVs, respectively, compared to patients without thrombosis (*p* < 0.05; Figure 1A,B; Figure S1A). No differences in MK-EV and PLT-EV proportions were observed between high- vs. low-risk PV patients (data not shown). Next, we explored the circulating PE-EVs (Figure S1B) and TF-EVs (Figure S1C). Notably, no significant difference was observed comparing patients with and without thrombosis history (data not shown). However, when cardiovascular risk factors (smoking, hypertension, diabetes, and dyslipidemia) were analyzed, the proportion of TF-EVs was significantly increased only in PV patients with hypertension (*p* < 0.05; Figure 1C) and positively correlated with age (*p* < 0.05; Figure 1D). In addition, only the proportion of TF-EVs was significantly correlated with the *JAK2V617F* VAF (r = 0.33, *p* < 0.05; Figure 1E). The proportion of PE-EVs was significantly increased in high-risk PV patients (*p* < 0.05; Figure 2A) and in PV patients with hypertension (*p* < 0.05; Figure 2B), and positively correlated with age (*p* < 0.05; Figure 2C).

Assessing marrow fibrosis, only PE-EVs were significantly increased in patients with fibrosis compared to patients without fibrosis (*p* < 0.05; Figure 2D).

Of note, no differences were observed when EV signatures were compared between *JAK2V617F*- and exon 12-mutated patients (data not shown).

Taken together, these results demonstrate that a distinct phenotype of the isolated EVs is associated with a history of thrombosis or marrow fibrosis of PV patients.

### 3.2. Isolated EVs of PV Patients Show a LPS-Associated Signature of Thrombosis

**Figure 3:**
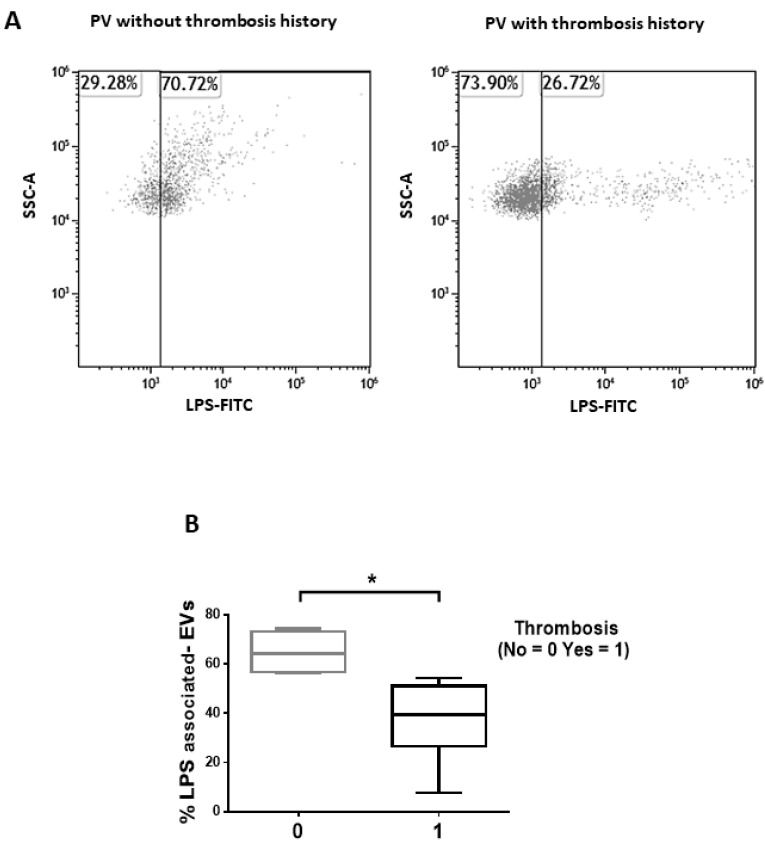
Proportion of LPS-associated EVs after isolation from the plasma of PV patients. (**A**) Representative dot-plots of LPS-associated EVs after isolation from the platelet-poor plasma of 1 PV patients with thrombosis history and 1 PV patient without thrombosis history. (**B**) Proportion of isolated LPS-associated EVs of PV patients with (*n* = 14) or without thrombosis (*n* = 24). Data are expressed as percentages and presented as min to max with median (Mann–Whitney test; * *p* < 0.05).

We then sought to analyze whether EVs isolated from the plasma of PV patients show a microbial-associated phenotype (LPS expression) and whether this phenotype might be related to outcome. Interestingly, flow cytometric analysis revealed that PV patients with thrombosis history showed reduced levels of LPS-associated EVs compared to patients without thrombosis (*p* < 0.05; Figure 3A,B). Of note, the expression of tetraspanins, namely CD81, CD63, and CD9, and the number/size of the isolated EVs were not significantly different between PV patients with or without thrombosis history (data not shown).

Collectively, these results demonstrate that the proportion of LPS-associated EVs distinguish PV patients with or without thrombosis history, being significantly lower in patients who experienced a thrombotic event.

### 3.3. Isolated EVs from PV Patients Carry Microbial DNA Signatures Associated with Thrombosis and Marrow Fibrosis

In parallel experiments, we analyzed whether microbial DNA traces might be detected in EVs isolated from the plasma of PV patients and whether they might depict a PV-specific signature of outcome. For this purpose, isolated EVs were subjected to 16S rRNA gene-based next-generation sequencing. A total of 941,230 sequence reads, with an average of 24,134 (±5059, SD) paired-end reads per sample, were obtained and analyzed.

**Figure 4:**
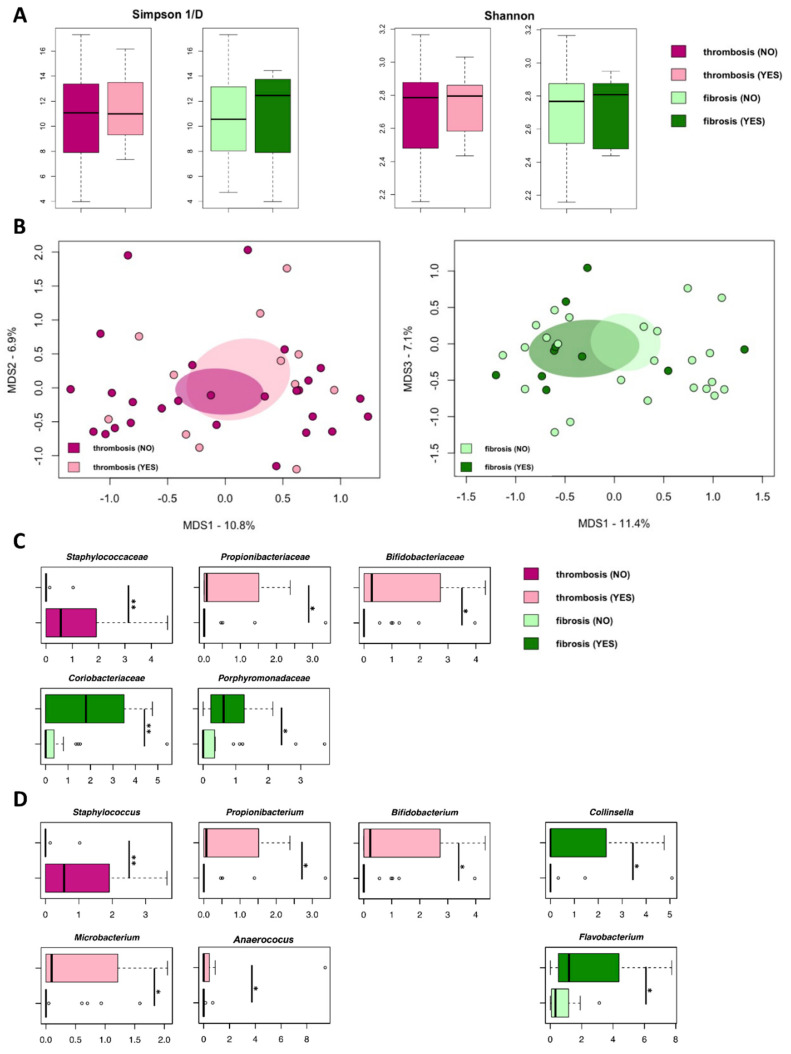
Microbial DNA signatures of thrombosis history and marrow fibrosis in EVs isolated from the plasma of PV patients. (**A**) Alpha diversity, estimated according to the inverse Simpson and Shannon indices, of the microbial DNA cargo of EVs isolated from PV patients stratified by the occurrence of thrombosis or marrow fibrosis. No significant differences were found (*p* ≥ 0.06, Wilcoxon rank-sum test). (**B**) Principal coordinates analysis (PCoA) based on Jaccard similarity between the genus-level profiles of EVs isolated from the plasma of PV patients, stratified as above. No significant segregation was found between study groups (*p* ≥ 0.3, PERMANOVA). Boxplots showing the relative abundance distribution of families (**C**) and genera (**D**) that were significantly differentially represented between PV patients with and without history of thrombosis or marrow fibrosis (* *p* < 0.05; ** *p* < 0.01; Wilcoxon rank-sum test).

According to the inverse Simpson and Shannon indices, no differences in alpha diversity were observed when comparing microbiomes from EVs from PV patients with or without thrombosis history or marrow fibrosis (Figure 4A). Similarly, principal coordinates analysis (PCoA) based on Jaccard similarity between the overall genus-level compositional microbiome profiles showed no segregation between PV patients with or without thrombosis history or marrow fibrosis (Figure 4B).

However, at the family level, EVs from PV patients were distinguished in the proportions of specific microbiome components, such as *Staphylococcaceae*, *Propionibacteriaceae*, and *Bifidobacteriaceae*, of which the former was increased in patients without thrombosis (*p* < 0.01, Wilcoxon rank-sum test) and the latter two were increased in patients with thrombosis (*p* < 0.05) (Figure 4C). As for marrow fibrosis, DNA enrichment in *Coriobacteriaceae* (*p* < 0.01) and *Porphyromonadaceae* (*p* < 0.05) was observed for EVs of PV patients with fibrosis when compared to patients without marrow fibrosis (Figure 4C).

Focusing on the behavior of the specific microbiome genera, *Staphylococcus* DNA was enriched in EVs from patients without thrombosis compared to patients with thrombosis (*p* < 0.01) (Figure 4D). Conversely, EVs from PV patients with thrombosis history were enriched in DNA assigned to Actinobacteria genera (i.e., *Propionibacterium*, *Bifidobacterium*, and *Microbacterium*) and *Anaerococcus*, compared to those without thrombosis (*p* < 0.05; Figure 4D). With regard to marrow fibrosis, DNA enrichment of *Collinsella* and *Flavobacterium* was significant compared to patients without marrow fibrosis (*p* < 0.05; Figure 4D). Collectively, EV-associated microbial DNA signatures of thrombosis history and marrow fibrosis emerged, including a marrow-fibrosis-specific increase in *Collinsella* and *Flavobacterium*, along with a thrombosis-specific depletion in staphylococci.

### 3.4. The Gut Microbiome Does Not Differ between PV Patients with or without Thrombosis History or Marrow Fibrosis

When fecal samples of PV patients were profiled by 16S rRNA gene-based next-generation sequencing, a total of 1,375,982 sequence reads, with an average of 36,210 (±10,765, sd) paired-end reads per sample, were obtained and analyzed.

**Figure 5:**
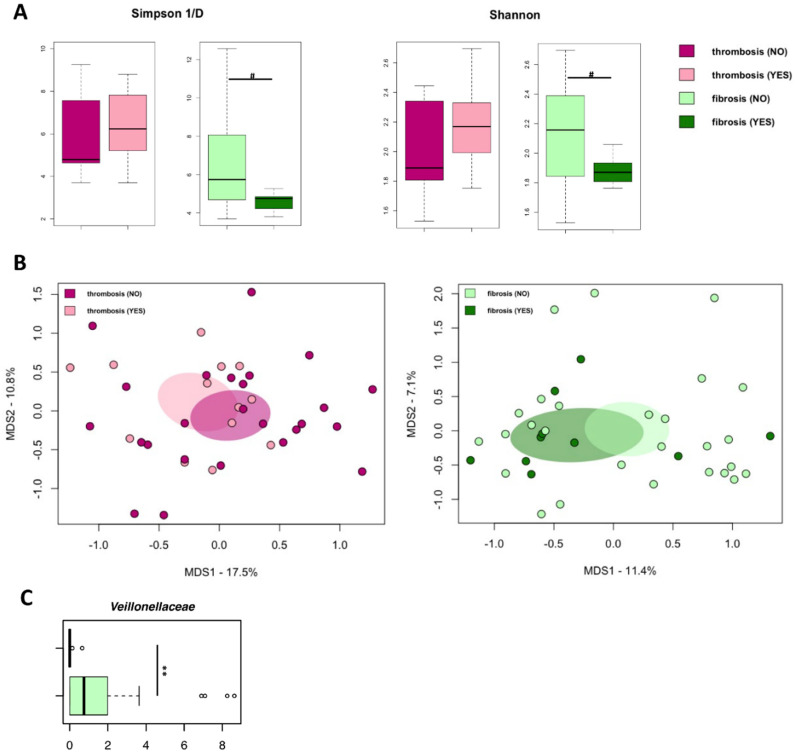
The gut microbiome of PV patients. (**A**) Alpha diversity estimated according to inverse Simpson (top) and Shannon (bottom) indices of PV patients with or without thrombosis history and marrow fibrosis. (**B**) Principal coordinates analysis (PCoA) based on Jaccard similarity between the genus-level microbiota profiles of PV patients with or without thrombosis history and marrow fibrosis. (**C**) Boxplot showing the relative abundance distribution of the genera that were differentially represented between PV patients with or without marrow fibrosis (# *p* < 0.05; ** *p* < 0.01; Wilcoxon rank-sum test).

Of note, no significant differences in biodiversity were observed when stratifying PV patients according to thrombosis or marrow fibrosis (Figure 5A). Consistently, Jaccard-based PCoA showed no segregation between PV patients with or without thrombosis history or marrow fibrosis (Figure 5B). The comparison of the microbiome profiles at the phylum, family, and genus level did not reveal any significant difference between PV patients with or without thrombosis history. Only PV patients with marrow fibrosis were depleted in *Veillonellaceae* with respect to those without fibrosis (*p* < 0.01; Figure 5C).

## 4. Discussion

Circulating EVs in combination with 16S sequencing have been previously investigated by different groups to evaluate microbial composition in different biofluids [42]. Notably, even though one of the critical limitations of the study was the lack of a control group of comparable age and hypertension, here, for the first time, we successfully identified PV-specific signatures of thrombosis history and marrow fibrosis based on the presence of EV-associated distinct phenotype and microbial layout.

Firstly, comparing patients with or without thrombosis history, we found that lower proportion of PLT-EVs and increased proportion of MK-EVs were associated with patients with thrombosis history. This might be due to the fact that PV patients with thrombosis history are heavy treated with anti-platelet and cytoreductive drugs, therefore, a reduced platelet activation might result in a decreased PLT-EV generation. Alternatively, the procoagulant state might favor the consumption/clearance of EVs of platelet origin. Conversely, we also found that the proportion of TF-EVs or PE-EVs were not significantly different between the two groups of patients.

Focusing on TF-EVs, our results are in contrast from those reported by Taniguchi et al. [25]. Whether this is due to the fact that their analysis referred to EVs derived from global MPN patients, and not PV patients only, remains a matter of speculation. However, consistent with this study, we demonstrated that the proportion of TF-EVs was correlated with the JAK2V617F VAF [25].

Secondly, with specific regard to thrombosis and taxonomic differences in DNA traces, EVs isolated from the plasma of PV patients with a history of thrombosis showed increased proportions of DNA assigned to several Actinobacteria members (*Propionibacterium*, *Bifidobacterium*, and *Microbacterium*) as well as *Anaerococcus*. Interestingly, *Propionibacterium* has been shown to mediate platelet activation and aggregation [43]. On the other hand, *Anaerococcus* strains isolated from the gut environment have been demonstrated to produce trimethylamine from choline in vitro, thus potentially contributing to the levels of the prothrombotic trimethylamine-N-oxide [14]. Although we do not know if EVs contain other potentially expressible microbial gene sequences or other microbially derived molecules, it is tempting to speculate that some of the detected microbial signatures could be directly involved in thrombotic events. Consistently, based on the study of circulating human microbiome, it has been previously demonstrated that patients with cardiovascular diseases (CVD) show an increase in microbial diversity and bacterial DNA concentration. Notably, at the phylum level, these authors observed a dominance of Actinobacteria in CVD patients. Therefore, we may hypothesize that the enrichment of Actinobacteria-derived DNA in EVs of PV patients with thrombosis history could be either the cause or consequence of thrombosis; a hypothesis that needs to be explored further [19,42,44]. Furthermore, PV patients with thrombosis history show also a depletion of DNA related to *Staphylococcus* genus, to which nosocomial pathogens belong but also commensal colonizers of human skin and mucus membranes. In particular, *Staphylococcus aureus* has recently been defined as a master manipulator of the human hemostatic system [45]. It should also be noted that the simultaneous presence of *Staphylococcus aureus* and LPS has been shown in vitro to induce homologous tolerance but heterologous priming [46], leading to the suppression of excessive proinflammatory mediator production of the adaptive/innate immune response. Although we do not know which species the staphylococcal DNA traces belong to, this potentially supports parallelism between reduced levels of LPS-associated EVs and *Staphylococcus* DNA-associated EVs in patients with thrombosis history, and suggests impaired immune responses, likely contributing to the maintenance of inflammation.

Interestingly, our results demonstrate that only DNA from Gram positive bacteria is differentially expressed in circulating EVs from patients with or without thrombosis history. However, even though we cannot discriminate whether EVs are of bacterial or human origin, we also found that the proportion of LPS-associated EVs distinguish PV patients with or without thrombosis history, being decreased in the presence of thrombosis. Bacterial LPS, the glycolipids found on the outer membrane of Gram-negative bacteria, is one of the links between the microbiome and hypercoagulability. LPS binds to toll-like receptors to activate endothelial cells and platelets, leading to activation of the coagulation cascade [47]. It might be argued that in patients with thrombosis history, the lower proportion of LPS-associated EVs might be due to the consumption/clearance of EVs in the procoagulant microenvironment. In addition, LPS plays a key role in platelet activation and PLT-EV production [48]. Since only activated platelets originate EVs [26], we can hypothesize that, in patients with thrombosis history, the lower levels of PLT-EVs might be related with the decreased proportion of LPS-associated EVs.

Finally, an increased proportion of PE-EVs was associated with marrow fibrosis, suggesting a role of PE-EVs in fibrotic marrow remodeling. It has been demonstrated that cellular stress, such as lipotoxic and/or endoplasmic reticulum stress conditions, induces rearrangements into cell membrane phospholipids and plays a crucial role in determining which lipids are released via EVs [49]. Therefore, we can hypothesize that the elevated proportions of PE-EVs are related to the inflammation and cellular stress of the bone marrow microenvironment in PV patients with fibrosis. One might also speculate that, since PV patients with marrow fibrosis are more likely to progress to myelofibrosis, the proportion of PE-EVs might be considered as a biomarker of fibrotic evolution and disease severity. Indeed, patients with a more aggressive disease (marrow fibrosis, older age, and hypertension) are associated to a higher proportion of PE-EVs.

Of note, we also identified potential bacterial biomarkers of marrow fibrosis in PV. Specifically, patients with marrow fibrosis showed an enrichment of EVs-associated *Collinsella* and *Flavobacterium* DNA. Even though these bacteria have never been associated with marrow fibrosis, based on our results, we might hypothesize their involvement in the genesis or resolution of marrow fibrosis.

As a last point, it should be noted that the gut microbiota profiles of PV patients with/without thrombotic events or marrow fibrosis did not differ from each other. While this further supports the role of circulating EVs as valuable biomarkers of disease, it could also suggest that other ecosystems, not (or not only) the gut, are the source of the microbial signatures of circulating EVs detected in the present study.

## 5. Conclusions

In conclusion, our data demonstrate that PV is associated with specific circulating EVs harboring distinctive eukaryotic and prokaryotic markers, with potential involvement in the major complication of PV, i.e., thrombosis, and as a predictive feature for MF transformation, i.e., marrow fibrosis. Interestingly, the microbial signature was specific to the circulating EVs, since the analysis of gut microbiota failed to identify difference between patients with or without thrombosis history or marrow fibrosis. Therefore, even though this needs to be confirmed in a larger cohort, for the first time, here, we described that, along with a distinct phenotype, bacterial DNA traces have been associated with circulating EVs in humans and, specifically, in PV patients. These findings support the role of EVs as biomarkers of liquid biopsy in PV. Further studies, possibly longitudinal and integrating other -omics approaches (e.g., metagenomics, metatranscriptomics, and metabolomics) as well as in vitro studies, are needed to validate these assumptions and deeply investigate the mechanisms behind selective bacterial DNA sorting in PV patients, and those of interaction with the host. Such studies should also include the characterization of other human-associated microbial communities (e.g., those of the mouth or skin) from which circulating EVs and/or their microbial signatures could derive. Finally, these data may contribute not only to refine the prognosis of PV patients but also to identify novel target (s) for personalized therapies focusing on thrombosis and marrow fibrosis prevention/treatment.

## Data Availability

Sequencing reads were deposited in the National Center for Biotechnology Information Sequence Read Archive (NCBI SRA) under the following Bioproject number: PRJNA737425 (https://www.ncbi.nlm.nih.gov/bioproject/PRJNA737425, 1 October 2021).

## Supplementary Materials

The following are available online at https://www.mdpi.com/article/10.3390/cancers13194968/s1, Table S1: Clinical and laboratory features of PV patients, Table S2: List of monoclonal antibodies and reagents according to EV subtype, Figure S1: Flow cytometry analysis of EVs after isolation from the plasma of PV patients.
