# Supplementary material for: A Specific Host/Microbial Signature of Plasma-Derived Extracellular Vesicles Is Associated to Thrombosis and Marrow Fibrosis in Polycythemia Vera
Source: Cancers (Basel). 2021 Oct 2;13(19):4968. doi: 10.3390/cancers13194968 (PMC8507916; doi:10.3390/cancers13194968)
Supplement: Supplementary file 1 [file cancers-13-04968-s001.zip › cancers-1371674-supplementary.pdf]

Table S1. Clinical and laboratory features of PV patients. Results are reported as median (range) or absolute number (percentage).

.	**PV (38 cases)**
**Age at study, years; median (range)**	63 (26-84)
**Males, no. (%)**	21 (55%)
**Hemoglobin, g/dL; median (range)**	17.7 (13.1-21.8)
**Leukocytes, × 10^9/L; median (range)**	8.9 (5.1-21.5)
**Platelets, × 10^9/L; median (range)**	370 (160-813)
**Hematocrit; median (range)**	55 (40.5-67.6)
**PV risk category, no. of patients (%)**	
High	27 (71%)
Low	11 (29%)
**Marrow fibrosis ≥1, no. of patients (%)**	10 (26%)
**Driver mutation (%)**	
JAK2 ^{V617F}	35 (92%)
JAK2 Exon 12	3 (8%)
**Cardiovascular risk factors, no. of patients (%)**	
smoking	10 (26%)
hypertension	24 (63%)
diabetes	4 (10.5%)
dyslipidemia	10 (26%)
**Thrombosis, no. of patients (%)**	14 (37%)
Arterial	5 (13%)
Venous	9 (24%)
**Thrombosis pre/at diagnosis, no. of patients (%)**	9 (24%)
**Thrombosis post-diagnosis, no. of patients (%)**	5 (13%)
**Bleeding, no. of patients (%)**	0
**Second cancer, no. of patients (%)**	3 (8%)
**Ongoing treatment, no. of patients (%)**	33 (87%)
Phlebotomy only	8 (21%)
Hydroxyurea only	6 (16%)
Phlebotomy and Hydroxyurea	25 (66%)
Anagrelide	0
Interferon	0
Ruxolitinib	0
Cardioaspirin	26 (68%)
**Ongoing antibiotic therapy, no. of patients (%)**	4 (11%)
**Ongoing probiotic treatment, no. of patients (%)**	5 (13%)

Table S2. List of monoclonal antibodies and reagents according to EV subtype.

EVs subtype	Identified as	Monoclonal antibodies/reagents
Megakaryocyte-EVs	CD61+/CD62P-	Anti-CD61 (Clone: SZ21; FITC-conjugated; Catalog number IM1758); Anti- CD62P (Clone: CLB-THROMB/6; PE-conjugated; Catalog number IM1759U). All antibodies from Beckman Coulter S.r.l.
Platelet-EVs	CD61+/CD62P+	Anti-CD61 (Clone: SZ21; FITC-conjugated; Catalog number IM1758); Anti- CD62P (Clone: CLB-THROMB/6; PE-conjugated; Catalog number IM1759U). All antibodies from Beckman Coulter S.r.l.
Tissue Factor-positive EVs	CD142+	Anti-CD142 (Clone: NY2; APC-conjugated; Catalog number 365206) from BioLegend
Phosphatidylethanolamine-positive EVs	Duramicin+	Duramycin-LC-Biotin (Catalog number 25690-100) from Polysciences, Inc. Anti- Biotin (Clone: Bio3-18E7; FITC-conjugated; Catalog number 130-113-852) from Miltenyi Biotec;
Tetraspanins-positive EVs	CD81+/CD9+/CD63+	Anti-CD81 (Clone: REA513; APC-conjugated; Catalog number 130-119-825) from Miltenyi Biotec; Anti-CD9 (Clone: REA1071; FITC-conjugated; Catalog number 130-118-806) from Miltenyi Biotec; Anti-CD63 (Clone: H5C6; PE-conjugated; Catalog number 130-100-158) from Miltenyi Biotec.
Lipopolysaccharide-positive EVs	LPS+	Anti-Lipopolysaccharide (LPS) (FITC-conjugated; Catalog number LAB526Ge82) from Cloud-Clone Corp.

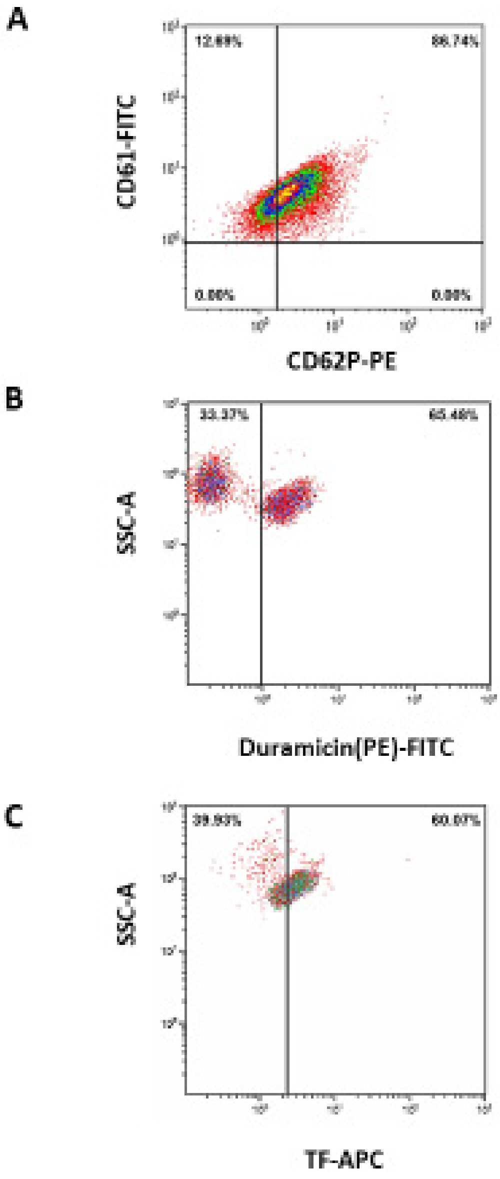

Figure S1. Flow cytometry analysis of EVs after isolation from the plasma of PV patients. (A, B and C) Representative dot-plots of MK- (CD61+/CD62P-), PLT- (CD61+/CD62P+), PE-(Duramicin+) and TF-EVs.
